# Exogenous Brassinolide Ameliorates the Adverse Effects of Gamma Radiation Stress and Increases the Survival Rate of Rice Seedlings by Modulating Antioxidant Metabolism

**DOI:** 10.3390/ijms252111523

**Published:** 2024-10-26

**Authors:** Yanting Lu, Bingkui Wang, Mengchao Zhang, Wenxin Yang, Mingming Wu, Jing Ye, Shenghai Ye, Guofu Zhu

**Affiliations:** Institute of Crop and Nuclear Technology Utilization, Zhejiang Academy of Agricultural Sciences, 198 Shi-Qiao Road, Hangzhou 310021, China; luyt@zaas.ac.cn (Y.L.); bingkuiwang2006@126.com (B.W.); zhangmc@zaas.ac.cn (M.Z.); yangwx@zaas.ac.cn (W.Y.); wumm@zaas.ac.cn (M.W.); yejingzj@163.com (J.Y.); yesh@zaas.ac.cn (S.Y.)

**Keywords:** brassinolide, gamma radiation, rice seedling, survival rate, protective effect, antioxidant metabolism

## Abstract

Gamma irradiation-based mutant creation is one of the most important methods for rice plant mutagenesis breeding and molecular biology research. Although median lethal dose irradiation severely damages rice seedlings, applying brassinolide (BR) can increase the survival rate of irradiated seedlings. In this study, we investigated the effects of soaking seeds in solutions containing different BR concentrations (0.001, 0.01, 0.1, 1.0, and 5.0 μmol/L) and then spraying the resulting seedlings twice with 0.1 μmol/L BR. The combined BR treatments markedly decreased the superoxide anion (O_2_^•−^), hydrogen peroxide (H_2_O_2_), and malondialdehyde contents but increased the chlorophyll content. An appropriate BR treatment of gamma-irradiated samples substantially increased the activities of the antioxidant enzymes superoxide dismutase, peroxidase, and ascorbate peroxidase as well as the proline, ascorbic acid, and glutathione contents in rice seedling shoots. The BR treatment also promoted the growth of seedlings derived from irradiated seeds and increased the shoot and root fresh and dry weights. Most notably, soaking seeds in 0.01 or 0.1 μmol/L BR solutions and then spraying seedlings twice with 0.1 μmol/L BR significantly increased the final seedling survival rate and decreased mutant loss. The study results suggest that exogenous BR treatments can protect rice seedlings from gamma irradiation stress by enhancing antioxidant metabolism.

## 1. Introduction

Rice is the most important food crop in China and also an important staple crop in Southeast Asia (He et al., 2023 and Li et al., 2023) [1,2]. In recent years, there has been a rapid increase in rice production in Africa (Yuan et al., 2024) [3]. In 2020, the rice-growing area in China reached 30.08 million hectares (18.28% of the global rice-growing area), with a total yield of 211.86 million tons (27.42% of the global yield). The yield per hectare in China is 7.043 tons, significantly higher than the global average of 4.694 tons per hectare (FAO 2024) [4]. In China, there have been notable achievements in rice production over the past 100 years, with five generations of elite varieties that have made significant contributions to ensuring global food security (Zhao et al., 2022) [5]. The most important reason for these achievements is the cultivation of new elite varieties supported by improvements to germplasm resources (Liu et al., 2022) [6]. The development of the Chinese economy has been accompanied by a change in the objectives of rice breeding programs. More specifically, in addition to maximizing yield and increasing disease and insect resistance, there is a growing emphasis on developing varieties that satisfy consumer requirements regarding quality as well as varieties with increased nitrogen fertilizer use efficiency, which may have beneficial effects on the environment (Zhao et al., 2022, Cai et al., 2023) [5,7]. Extreme climate changes have increased the demand for rice varieties that are tolerant to high temperatures and drought (Munson et al., 2021 and Dong et al., 2023) [8,9]. Thus, high-quality germplasm resources that are resistant or highly tolerant to biotic and abiotic stresses (i.e., diseases and/or insect pests, heat, drought, salinity, alkalinity, and nutrient deficiency) and able to efficiently use nitrogen while producing grains with a desirable taste will need to be identified and exploited (Sagehashi et al., 2022; He et al., 2022; Zheng et al., 2024) [10,11,12]. Irradiating seeds to create mutants is an important method for modifying germplasm resources for rice breeding; this method has been successfully used in China over the past 70 years and will continue to play a key role in rice breeding programs (Lu et al., 2017 and 2024) [13,14].

An appropriate gamma-ray dose for the irradiation of rice seeds can induce various changes in DNA and chromosomal structure while also affecting gene expression, resulting in countless mutants and new germplasm resources (Mounir et al., 2022) [15]. However, the irradiation of rice seeds also results in cell damage and substantially increases reactive oxygen species (ROS) levels in seeds and seedlings (Lu et al., 2024) [14]. Generally, the moisture content of rice seeds is approximately 14%. A radiation treatment of rice seeds will induce the ionization of water molecules (H_2_O^+^) and generate numerous radicals (e.g., H^+^ and OH^−^). The production of secondary ROS due to water radiolysis, such as the hydroxyl radical (OH^−^), hydroperoxide radical (HO_2_^−^), hydrogen peroxide (H_2_O_2_), and superoxide anion (O_2_^•−^), results in lipid peroxidation, protein and nucleic acid modifications, chromosomal aberrations, and carbohydrate and pigment damages, which may be harmful to plants (Archanachai et al., 2021, Liu et al., 2021) [16,17]. Thus, radiation-induced mutations may enhance germplasm resources for breeders, but radiation is also an abiotic stressor of plants (Colak et al., 2021 and Kurt-Celebi et al., 2023) [18,19]. We recently revealed the importance of ROS generation and the corresponding antioxidant activities for plant mutagenesis (Lu et al., 2024) [14].

In plants, brassinolide (BR), a novel plant hormone isolated from the pollen of *Brassica napus* L., plays a significant role in cell division, protein synthesis, photosynthetic reactions, gene expression, and stress responses (Bhandari and Nailwal 2020 and Ahmed et al., 2023) [20,21]. Many recent studies examined BR response mechanisms and the contribution of BR to plant resistance to temperature extremes, drought, waterlogging, salinity, heavy metals, pesticides, and biotic stresses (Lv et al., 2022; Ye et al., 2023; Chen et al., 2024; Wu et al., 2024; Mu et al., 2022; Hussain et al., 2020; Duhan et al., 2024) [22,23,24,25,26,27,28]. The physiological and molecular mechanism underlying the regulatory effects of BR in plants under stress conditions is currently a major topic of interest among researchers. Tanveera et al. (2018) [29] reported that BR can improve plant growth and development under saline conditions by functioning as a signaling compound affecting different metabolic and physiological processes. Wu et al. (2024) [25] observed that BR can protect *Pinellia ternata* from waterlogging stress by increasing the antioxidant capacity and promoting the accumulation of soluble sugars and active ingredients. Moreover, BR can mitigate the adverse effects of shading on tea yield by increasing total amino acid, geraniol, chlorophyll, and carotenoid contents (Chen et al., 2023) [30]. Notably, BR can increase strawberry fruit yield and quality in subtropical regions by promoting root and shoot growth and increasing the number of flowers as well as fruit sugar and total soluble solids contents (Khatoon et al., 2023) [31]. However, whether BR can alleviate stress-related damages during radiation mutagenesis-based breeding of rice has not been investigated. To the best of our knowledge, this is the first study to assess whether plant regulators, such as BR, can increase the rice seedling survival rate by decreasing radiation stress-induced damages. The findings of this study may provide the theoretical basis and technical insights relevant to optimizing the application of BR and other plant growth regulators to enhance the survival of mutagenized seedlings and decrease mutant losses.

## 2. Results

### 2.1. Rice Seed Germination, Seedling Emergence, Seedling Growth, and Seedling Morphology

**Figure 1:**
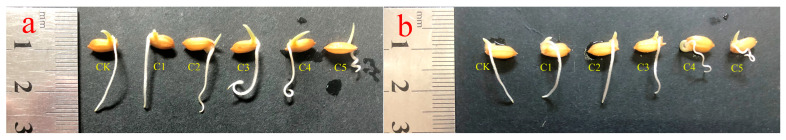
Effects of different BR concentrations on the root and shoot growth of two rice varieties. (**a**) Zhe 1613. (**b**) Zhejing 100. From left to right: CK sample and samples soaked in 0.001 (C1), 0.01 (C2), 0.1 (C3), 1.0 (C4), and 5.0 (C5) μmol/L BR solutions.

Soaking seeds in solutions with different BR concentrations affected the germination rate (Table 1). For Zhe 1613 (*indica* rice variety), there were no significant differences in the germination rates of the seeds treated with C1, C2, C3, and C4 and the control seeds. However, the C5 treatment significantly decreased the germination rate. For Zhejing 100 (*japonica* rice variety), the effect of different BR treatments on the germination rate is the same as that of Zhe 1613. Soaking seeds in solutions with different BR concentrations significantly affected seed root morphology during germination, with high BR concentrations resulting in clearly distorted roots (Figure 1 and Figure S1).

**Figure 2:**
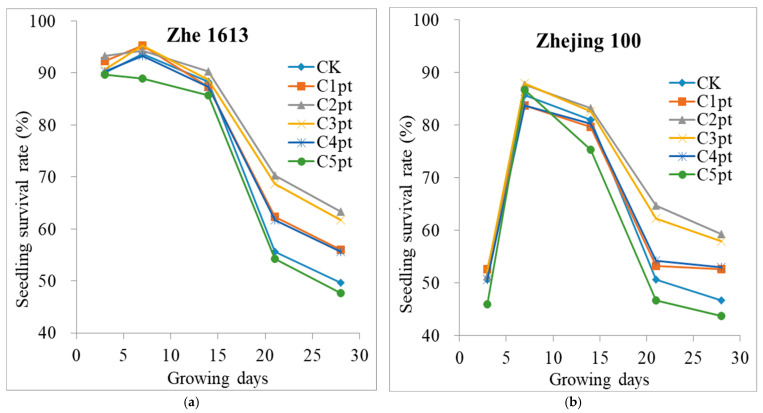
Effects of different BR treatment combinations on the survival rate of irradiated rice seedlings. (**a**) Zhe 1613. (**b**) Zhejing 100. The treatments were as follows: soaking in 0 (CK), 0.001 (C1pt), 0.01 (C2pt), 0.1 (C3pt), 1.0 (C4pt), and 5.0 (C5pt) μmol/L BR solutions and two post-sowing spray applications (0.1 μmol/L BR). Values represent the mean of three replicates ± SE.

After treating seeds of the two rice varieties with the median lethal dose (LD_50_) of gamma rays, the survival rates of the resulting seedlings peaked at 7 days post-emergence, after which they decreased on days 14, 21, and 28 (Figure 2 and Figure S2). Soaking seeds in solutions with different BR concentrations increased the seedling survival rate (Figure S2).

However, the effect was limited, mostly because the seeds had been irradiated. Interestingly, soaking the seeds in a solution with an appropriate BR concentration and then spraying the resulting seedlings twice with a 0.1 μmol/L BR solution significantly increased the seedling survival rate (Figure 2 and Table 2).

The different combined BR treatments significantly increased the final seedling survival rate (Figure 2 and Table 2). For Zhe 1613, the C1pt, C2pt, C3pt, and C4pt treatments significantly increased (*p* ≤ 0.05) the seedling survival rate by 12.68%, 27.36%, 24.14%, and 12.07%, respectively (relative to the control seedling survival rate). Among these treatments, C2pt and C3pt increased the seedling survival rate significantly more than the C1pt, C4pt, and C5pt treatments. Although the seedling survival rate was 4.02% lower for the C5pt treatment than for the control treatment, this difference was not significant. For Zhejing 100, the C1pt, C2pt, C3pt, and C4pt treatments significantly increased the seedling survival rate by 12.85%, 26.98%, 24.20%, and 13.49%, respectively (relative to the control seedling survival rate). Of these treatments, C2pt and C3pt increased the seedling survival rate significantly more than C1pt, C4pt, and C5pt. The final survival rate was lower for C5pt than for the control treatment, but the difference was not significant. These results suggest that soaking rice seeds in a solution with a high BR concentration may adversely affect seedling survival.

### 2.2. Seedling Growth Parameters

**Figure 3:**
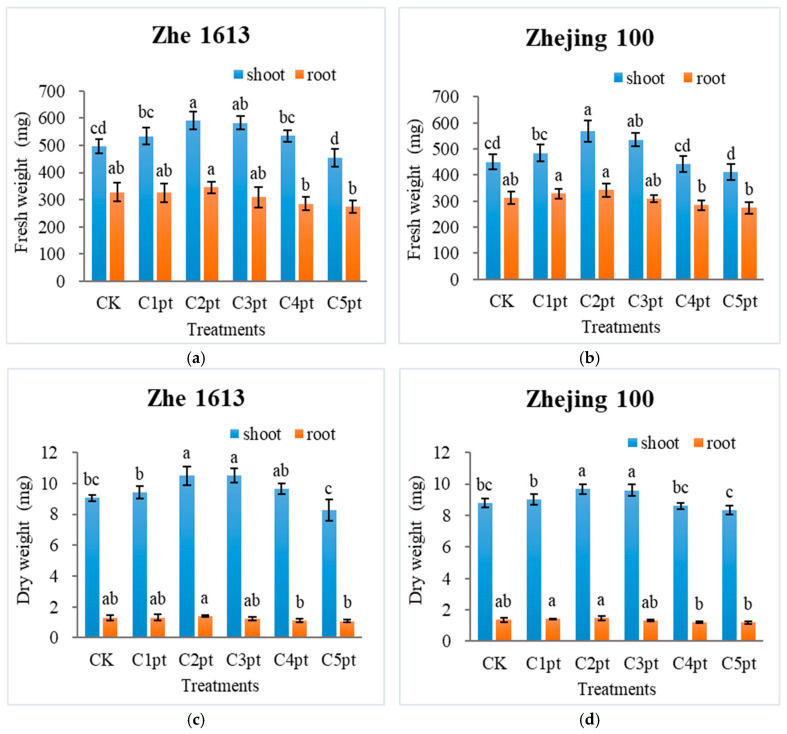

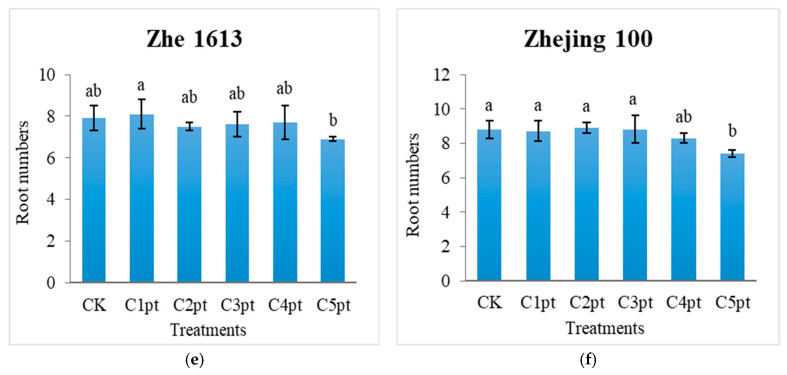
Effects of different BR treatment combinations on rice seedling shoot and root fresh weight (**a**,**b**) and dry weight (**c**,**d**) as well as the number of roots (**e**,**f**). The treatments were as follows: soaking in 0 (CK), 0.001 (C1pt), 0.01 (C2pt), 0.1 (C3pt), 1.0 (C4pt), and 5.0 (C5pt) μmol/L BR solutions and two post-sowing spray applications (0.1 μmol/L BR). Different letters indicate significant differences among treatments at the 0.05 level (Duncan). Values represent the mean of three replicates ± SE.

Figure 3 presents the effects of different BR treatment combinations on rice seedling shoot and root fresh and dry weights. For Zhe 1613, the C1pt, C2pt, and C3pt treatments increased the shoot fresh and dry weights (Figure 3a,c). More specifically, C2pt and C3pt significantly increased (*p* ≤ 0.05) the shoot fresh weight by 19.23% and 17.28%, respectively (relative to the control shoot fresh weight). The changes in the shoot dry weight were similar to the changes in the shoot fresh weight. Similar trends in the shoot fresh and dry weights were observed for Zhejing 100 treated with BR (Figure 3b,d). The Zhe 1613 root fresh weight was higher for the C2pt treatment than for the other BR treatments and the control treatment. For Zhejing 100, the root fresh weight was higher after the C1pt and C2pt treatments than after the control treatment. For both varieties, the root fresh weight was significantly higher for the C1pt and C2pt treatments than for the C4pt and C5pt treatments. The trends in the root dry weight were consistent with the trends in the root fresh weight.

There were no significant differences in the effects of different BR treatment combinations on seedling height and root length for both rice varieties (Figure S3), but there were significant differences in the number of roots. For Zhe 1613, the C1 treatment resulted in significantly more roots than the C5 treatment, but the effect of the C1 treatment did not differ significantly from the effects of the other treatments and CK (Figure 3e). Similar trends were observed for Zhejing 100 (Figure 3f).

### 2.3. Chlorophyll Content

**Figure 4:**
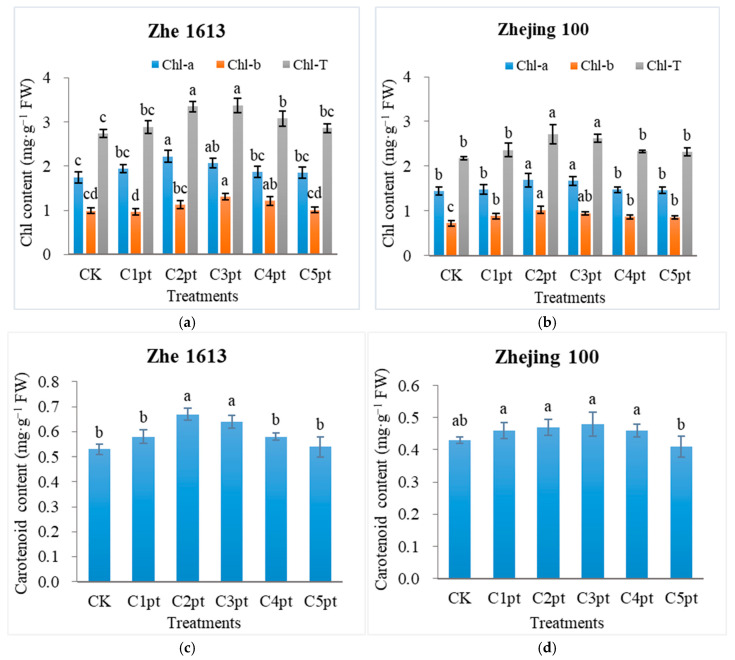
Effects of different BR treatment combinations on rice seedling chlorophyll (**a**,**b**) and carotenoid (**c**,**d**) contents. The treatments were as follows: soaking in 0 (CK), 0.001 (C1pt), 0.01 (C2pt), 0.1 (C3pt), 1.0 (C4pt), and 5.0 (C5pt) μmol/L BR solutions and two post-sowing spray applications (0.1 μmol/L BR). Different letters indicate significant differences among treatments at the 0.05 level (Duncan). Values represent the mean of three replicates ± SE.

Soaking irradiated seeds in solutions with different BR concentrations prior to sowing and then spraying the resulting seedlings twice with a BR solution increased the chlorophyll content of rice seedlings. For Zhe 1613, the chlorophyll *a* content increased by 10.86%, 26.86%, 18.29%, 6.86%, and 5.71% after the C1pt, C2pt, C3pt, C4pt, and C5pt treatments, respectively (relative to the control chlorophyll *a* content). The chlorophyll *a* content was significantly higher for the C2pt treatment than for the control, C1pt, C4pt, and C5pt treatments, but there were no significant differences in the effects of the C2pt and C3pt treatments (Figure 4a). For Zhejing 100, the chlorophyll *a* content increased by 2.78%, 17.36%, 15.97%, 2.08%, and 1.39% following the C1pt, C2pt, C3pt, C4pt, and C5pt treatments, respectively (relative to the control chlorophyll *a* content). The C2pt and C3pt treatments increased the chlorophyll *a* content significantly more than the control, C1pt, C4pt, and C5pt treatments (Figure 4b).

Different BR treatment combinations also substantially affected the seedling chlorophyll *b* content of the two rice varieties. For Zhe 1613, the chlorophyll *b* content increased by 14.14%, 32.32%, 22.22%, and 2.02% after the C2pt, C3pt, C4pt, and C5pt treatments, respectively (relative to the control chlorophyll *b* content), with the C3pt and C4pt treatments resulting in significantly greater increases than the control, C1pt, and C5pt treatments (Figure 4a). For Zhejing 100, the chlorophyll *b* content increased by 22.22%, 41.67%, 31.94%, 20.83%, and 19.44% following the C1pt, C2pt, C3pt, C4pt, and C5pt treatments, respectively (relative to the control chlorophyll *b* content). The C2pt treatment resulted in a significantly higher chlorophyll *b* content than the control, C1pt, C4pt, and C5pt treatments; however, there were no significant differences between the effects of the C2pt and C3pt treatments (Figure 4b).

Different BR treatment combinations also significantly altered the total chlorophyll content of rice seedlings. For both varieties, the C2pt and C3pt treatments resulted in the highest total chlorophyll contents in seedlings. In addition, the carotenoid content of Zhe 1613 seedlings was significantly higher after the C2pt and C3pt treatments than after the control treatment and the other BR treatments (Figure 4c). For Zhejing 100, the carotenoid content was significantly lower for the C5pt treatment than for the control treatment and the other BR treatments. There were no significant differences between the other BR treatments and the control treatment in terms of the carotenoid content (Figure 4c,d).

### 2.4. Lipid Peroxidation

**Figure 5:**
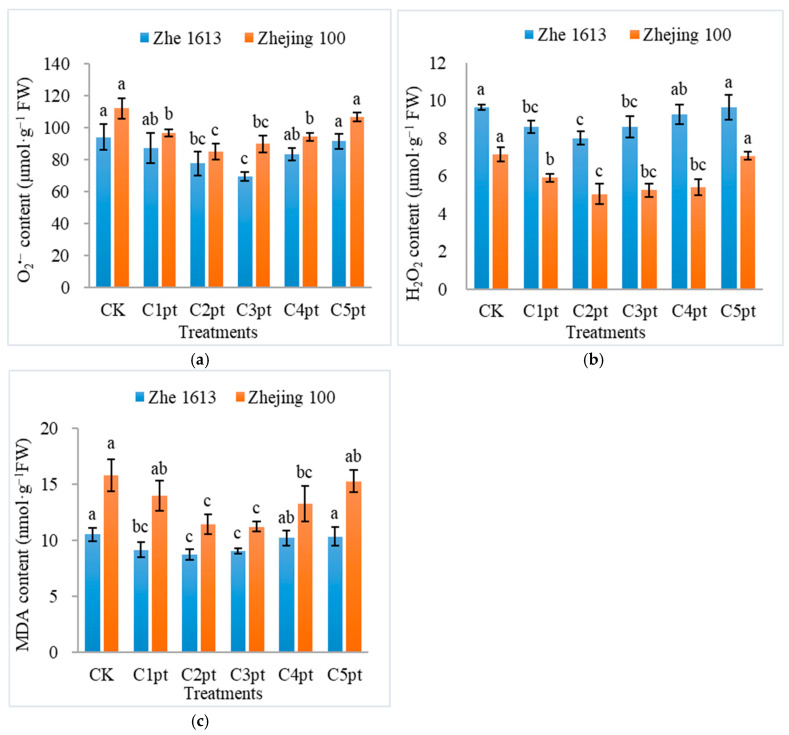
Effects of different BR treatment combinations on rice seedling O_2_^•−^ (**a**), H_2_O_2_ (**b**), and MDA (**c**) contents. The treatments were as follows: soaking in 0 (CK), 0.001 (C1pt), 0.01 (C2pt), 0.1 (C3pt), 1.0 (C4pt), and 5.0 (C5pt) μmol/L BR solutions and two post-sowing spray applications (0.1 μmol/L BR). Different letters indicate significant differences among treatments at the 0.05 level (Duncan). Values represent the mean of three replicates ± SE.

Different BR treatment combinations significantly affected the seedling O_2_^•−^ content for both rice varieties. For Zhe 1613, the O_2_^•−^ content decreased by 7.12%, 17.44%, 26.06%, 11.34%, and 2.67% after the C1pt, C2pt, C3pt, C4pt, and C5pt treatments, respectively (relative to the control O_2_^•−^ content). The C3pt treatment resulted in a significantly lower O_2_^•−^ content than the control, C1pt, C4pt, and C5pt treatments, but there were no significant differences in the effects of the C3pt and C2pt treatments (Figure 5a). For Zhejing 100, the O_2_^•−^ content decreased by 13.51%, 23.96%, 19.80%, 15.81%, and 4.71% following the C1pt, C2pt, C3pt, C4pt, and C5pt treatments, respectively (relative to the control O_2_^•−^ content). The C2pt treatment resulted in a significantly lower O_2_^•−^ content than the control, C1pt, C4pt, and C5pt treatments, but its effect did not differ significantly from that of the C3pt treatment (Figure 5a).

The BR treatment also decreased the H_2_O_2_ content in rice seedlings. For Zhe 1613, the H_2_O_2_ content decreased by 10.72%, 16.88%, 10.83%, and 3.75% after the C1pt, C2pt, C3pt, and C4pt treatments, respectively (relative to the control H_2_O_2_ content). The C2pt treatment resulted in a significantly lower H_2_O_2_ content than the control, C4pt, and C5pt treatments (Figure 5b). For Zhejing 100, the H_2_O_2_ content decreased by 17.29%, 29.42%, 26.62%, 24.10%, and 1.28% following the C1pt, C2pt, C3pt, C4pt, and C5pt treatments, respectively (relative to the control H_2_O_2_ content). The H_2_O_2_ content was significantly lower for the C2pt treatment than for the control, C1pt, and C5pt treatments (Figure 5b).

Significant changes in the seedling malondialdehyde (MDA) content were detected in response to the BR treatments. For Zhe 1613, the MDA content of rice seedlings treated with C1pt, C2pt, C3pt, C4pt, and C5pt decreased by 13.11%, 17.09%, 14.06%, 2.85%, and 1.71%, respectively (relative to the control MDA content). The MDA content was significantly lower for the C2pt and C3pt treatments than for the control, C4pt, and C5pt treatments (Figure 5c). For Zhejing 100, the MDA content of rice seedlings treated with C1pt, C2pt, C3pt, C4pt, and C5pt decreased by 11.48%, 27.28%, 28.87%, 16.05%, and 3.17%, respectively (relative to the control MDA content). The MDA content was significantly lower for the C2pt and C3pt treatments than for the control, C1pt, and C5pt treatments (Figure 5c).

### 2.5. Antioxidant Contents

**Figure 6:**
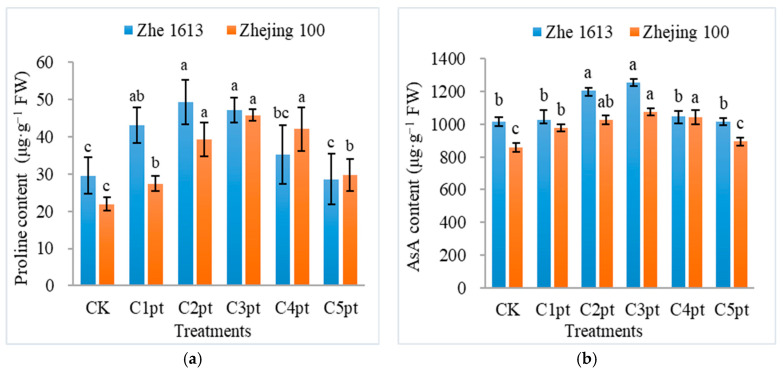

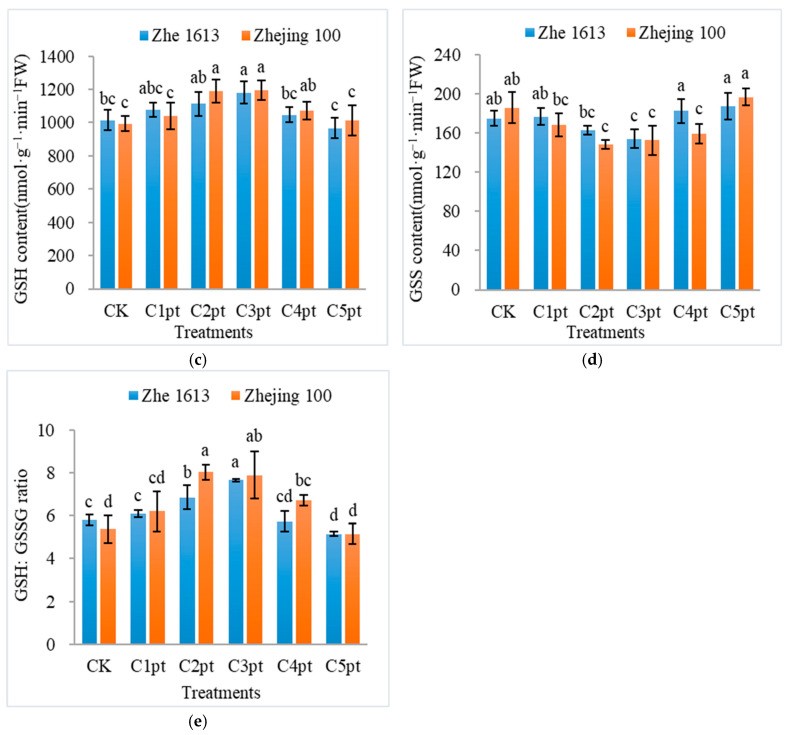
Effects of different BR treatment combinations on rice seedling proline (**a**), AsA (**b**), GSH (**c**), and GSSG (**d**) contents, as well as the GSH: GSSG ratio (**e**). The treatments were as follows: soaking in 0 (CK), 0.001 (C1pt), 0.01 (C2pt), 0.1 (C3pt), 1.0 (C4pt), and 5.0 (C5pt) μmol/L BR solutions and two post-sowing spray applications (0.1 μmol/L BR). Different letters indicate significant differences among treatments at the 0.05 level (Duncan). Values represent the mean of three replicates ± SE.

The effects of different BR treatment combinations on antioxidant contents were investigated. The proline content of rice seedlings changed significantly after the BR treatment. For Zhe 1613, the proline content of seedlings treated with C1pt, C2pt, C3pt, and C4pt increased by 45.83%, 66.64%, 59.24%, and 18.79%, respectively (relative to the control proline content). The proline content was significantly higher for the C2pt and C3pt treatments than for the control, C4pt, and C5pt treatments. Although the proline content appeared to be lower for the C5pt treatment than for the control treatment, the difference was not significant (Figure 6a). For Zhejing 100, the proline content of seedlings treated with C1pt, C2pt, C3pt, C4pt, and C5pt increased by 24.94%, 79.72%, 109.32%, 92.42%, and 35.86%, respectively (relative to the control proline content). The proline content was significantly higher for the C2pt, C3pt, and C4pt treatments than for the control, C1pt, and C5pt treatments (Figure 6a). The changes in the ascorbic acid (AsA) content of the two rice varieties treated with BR were similar to the proline content changes, with C2pt and C3pt producing the best results (Figure 6b).

The reduced glutathione (GSH) levels in the shoots markedly increased after the combined BR treatments, which was in contrast to the clear decrease in the oxidized glutathione (GSSG) content. For Zhe 1613, the GSH content of seedlings treated with C1pt, C2pt, C3pt, and C4pt increased by 6.22%, 9.63%, 16.28%, and 3.20%, respectively (relative to the control GSH content). The GSH content was significantly higher for the C3pt treatment than for the control, C4pt, and C5pt treatments. The GSH content was lower for the C5pt treatment than for the control treatment, but the difference was not significant (Figure 6c). For Zhejing 100, the GSH content of seedlings treated with C1pt, C2pt, C3pt, C4pt, and C5pt increased by 4.67%, 19.98%, 20.18%, 7.83%, and 2.03%, respectively (relative to the control GSH content). The GSH content was significantly higher for the C2pt, C3pt, and C4pt treatments than for the control, C1pt, and C5pt treatments (Figure 6c). The BR treatments generally had the opposite effects on the seedling GSSG and GSH contents for both rice varieties (Figure 6d). For Zhe 1613, the GSH:GSSG ratio was significantly higher for the C2pt and C3pt treatments than for the control treatment and the other BR treatments (Figure 6e). For Zhejing 100, the GSH:GSSG ratio was also significantly higher for the C2pt and C3pt treatments than for the control treatment and the other BR treatments, but the difference between the effects of the C3pt and C4pt treatments was not significant (Figure 6e).

### 2.6. Antioxidant Enzyme Activities

**Figure 7:**
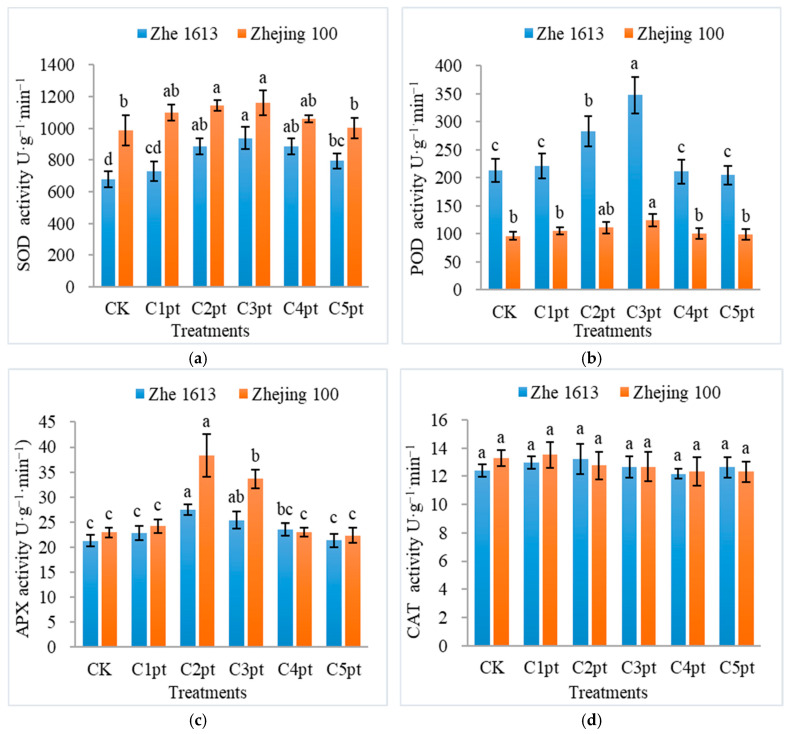
Effects of different BR treatment combinations on rice seedling SOD (**a**), POD (**b**), APX (**c**), and CAT (**d**) activities. The treatments were as follows: soaking in 0 (CK), 0.001 (C1pt), 0.01 (C2pt), 0.1 (C3pt), 1.0 (C4pt), and 5.0 (C5pt) μmol/L BR solutions and two post-sowing spray applications (0.1 μmol/L BR). Different letters indicate significant differences among treatments at the 0.05 level (Duncan). Values represent the mean of three replicates ± SE.

The effects of different BR treatment combinations on antioxidant enzyme activities were explored. According to the results, the BR treatment significantly increased superoxide dismutase (SOD), peroxidase (POD), and ascorbate peroxidase (APX) activities (relative to the control levels) (Figure 7). For Zhe 1613, the SOD activity in rice seedlings treated with C1pt, C2pt, C3pt, C4pt, and C5pt increased by 7.25%, 30.52%, 38.16%, 30.51%, and 17.10%, respectively (relative to the control SOD activity). The SOD activity was significantly higher for the C3pt treatment than for the control and C5pt treatments, but the effect of the C3pt treatment did not differ significantly from the effects of the C2pt and C4pt treatments (Figure 7a). For Zhejing 100, the SOD activity of seedlings treated with C1pt, C2pt, C3pt, C4pt, and C5pt increased by 11.26%, 15.99%, 17.79%, 7.24%, and 1.47%, respectively (relative to the control SOD activity). The SOD activity was significantly higher for the C2pt and C3pt treatments than for the control and C5pt treatments (Figure 7a). The POD activity in the Zhe 1613 seedlings treated with C1pt, C2pt, and C3pt increased by 3.24%, 32.80%, and 62.76%, respectively (relative to the control POD activity). The POD activity was significantly higher for the C3pt treatment than for the control treatment and the other BR treatments. The POD activity in the C4pt- and C5pt-treated seedlings decreased by 1.20% and 4.22%, respectively (relative to the control POD activity), but these changes were not significant (Figure 7b). For Zhejing 100, the POD activity of the seedlings treated with C1pt, C2pt, C3pt, C4pt, and C5pt increased by 8.76%, 15.05%, 29.10%, 3.81%, and 2.4%, respectively (relative to the control POD activity). The POD activity was significantly higher for the C3pt treatment than for the other treatments, except for C2pt (Figure 7b). The BR treatments increased the APX activity. For Zhe 1613, the APX activity of seedlings treated with C1pt, C2pt, C3pt, C4pt, and C5pt increased by 7.50%, 29.22%, 19.44%, 10.56%, and 0.30%, respectively (relative to the control APX activity). The APX activity was significantly higher for the C2pt treatment than for the control, C1pt, C4pt, and C5pt treatments, but there was no significant difference between the effects of the C2pt and C3pt treatments (Figure 7c). For Zhejing 100, the APX activity of seedlings treated with C1pt, C2pt, C3pt, and C4pt increased by 5.35%, 67.13%, 46.50%, and 0.12%, respectively (relative to the control APX activity). The APX activity was significantly higher for the C2pt and C3pt treatments than for the control, C1pt, C4pt, and C5pt treatments (Figure 7c). None of the BR treatments significantly affected the catalase (CAT) activity in seedlings for both varieties (Figure 7d).

## 3. Discussion

Radiation mutagenesis is one of the most important techniques used by crop breeders. The gamma irradiation of rice seeds can mutate genes and produce various mutants, some of which may be relevant to plant breeding (Lu et al., 2024) [14]. However, gamma irradiation at LD_50_ results in a final seedling survival rate of only approximately 50%. Some of the dead seedlings may carry genetic mutations that are highly useful for plant breeding and molecular biology research. For gamma irradiation, LD_50_ is considered to be the ideal dose. Thus, methods for increasing the survival rate of rice seedlings treated with this dose are needed. Although soaking irradiated rice seeds in a solution with an appropriate BR concentration can significantly increase the survival rate of the resulting seedlings (Table S1 and Figure S2), the survival rate will need to be increased further for practical breeding work. Therefore, we developed a combined BR treatment in which seeds are soaked in solutions containing different BR concentrations, and then the resulting seedlings are sprayed twice with a 0.1 μmol/L BR solution. The results of the current study indicate that this combined BR treatment is effective (Figure 2 and Table 2). More specifically, the seedling survival rates were highest for the C2pt and C3pt treatments. These two treatments also resulted in the highest shoot and root fresh and dry weights. However, whether 0.1 μmol/L BR is the optimal concentration for spraying remains to be determined. Mu et al. (2022) [26] reported that soaking rice seeds in a 0.1 mg/L BR solution significantly increases seedling growth under salt-stress conditions. Shang et al. (2023) [32] observed that a 1.0 mg/L BR priming treatment of rice seeds was ideal for enhancing rice seedling growth under saline conditions. However, there are no reports describing the effects of exogenous BR treatments on irradiated rice seed germination and seedling growth. Zhao et al. (2016) [33] showed that spraying rice seedlings with a solution containing a low BR concentration can promote root growth, but treatments with high BR concentrations inhibit root growth. In the current study, soaking seeds in a solution with a high BR concentration resulted in abnormal root growth (Figure 1 and Figure S2). It is possible that BR mediated the free IAA level in rice by regulating the accumulation of IAA amide hydrolase (Zhao et al., 2016) [33].

The gamma irradiation of rice seeds substantially increased ROS levels in both varieties (Figure 5). The combined BR treatments decreased the O_2_^•−^, H_2_O_2_, and MDA contents but simultaneously increased the chlorophyll *a* and *b*, total chlorophyll, and carotenoid contents, especially the C2pt and C3pt treatments. In accordance with these findings, earlier studies showed that BR can increase the total chlorophyll and carotenoid contents in maize and *P. ternata* under drought stress, suggesting that BR treatments increase the photosynthetic capacity by stabilizing the thylakoid membrane and increasing the photosynthetic pigment content in maize and *P. ternata* seedlings under drought conditions (Wang et al., 2023; Chen et al., 2024) [24,34] and in rice seedlings exposed to salinity stress (Mu et al., 2022) [26].

The protective effects of the combined BR treatment may be due to the associated increases in antioxidant enzyme activities and antioxidant contents. The gamma irradiation of rice seeds increased the O_2_^•−^ and H_2_O_2_ contents and stimulated plant antioxidant enzymes to eliminate ROS, thereby enabling the seedlings to continue to grow (Shehata et al., 2022) [35]. SOD, POD, CAT, and APX are four of the main antioxidant enzymes that protect plants from oxidative damage caused by radiation and other abiotic stresses (Liu et al., 2021 and Zhanassova et al., 2021) [17,36]. Although antioxidant enzyme activities decreased in irradiated seedlings because of the damage caused by the gamma irradiation, the BR treatment increased the activities of key antioxidant enzymes (Figure 7). Interestingly, the BR treatments had no significant effects on CAT activities in both varieties, but they markedly increased SOD, POD, and APX activities. The C2pt and C3pt treatments generally increased SOD, POD, and APX activities more than the other treatments. This is consistent with the results of earlier studies on rice seedlings under saline conditions (Mu et al., 2022) [22] and maize seedlings exposed to drought stress (Wang et al., 2023) [34]. Li et al. (2019) [37] reported that exogenous BR treatments result in considerable increases in the expression of two BR synthesis-related genes (*D2* and *D11*), thereby promoting rice growth in saline environments. Increases in antioxidant enzyme activities may be related to the restorative effects of BR on plant vitality, but the precise underlying metabolic mechanism will need to be identified and characterized.

Antioxidants, including proline, AsA, and GSH, are important for scavenging excessive free radicals and alleviating the adverse effects of abiotic stress. In the present study, the gamma irradiation of seeds significantly increased the O_2_^•−^ and H_2_O_2_ contents in rice seedlings, but the application of exogenous BR increased the proline, AsA, and GSH contents as well as the GSH:GSSG ratio in rice seedlings. Moreover, the C2pt and C3pt treatments had the most positive effects on SOD, POD, and APX activities. In accordance with these observations, Chen et al. (2024) [24] revealed BR protects *P. ternata* from the harmful effects of drought stress, while Mu et al. (2022) [26] determined BR increases the resistance of rice seedlings to NaCl stress. These findings imply BR treatments likely enhance the AsA–GSH cycle (Chen et al., 2024) [24], but the underlying mechanism will need to be analyzed more thoroughly in future studies.

In conclusion, soaking rice seeds in a solution with an appropriate BR concentration and then spraying the seedlings twice with a 0.1 μmol/L BR solution can significantly decrease O_2_^•−^, H_2_O_2_, and MDA contents while also increasing chlorophyll and antioxidant contents as well as antioxidant enzyme activities in seedlings. Furthermore, this combined BR treatment can significantly increase the rice seedling survival rate, thereby decreasing the loss of mutants. Both 0.01 and 0.1 μmol/L BR may be ideal concentrations for the seed soaking treatment.

## 4. Materials and Methods

### 4.1. Plant Materials and Radiation Treatments

Seeds of *indica* rice variety Zhe 1613 and *japonica* rice variety Zhejing 100 were obtained from the Institute of Crop and Nuclear Technology Utilization, Zhejiang Academy of Agricultural Sciences, Hangzhou, China. Each seed sample (100 g) was packed in a polyethylene box and irradiated at room temperature (20 ± 1 °C) using a cesium-137 (^137^Cs) source (Zhejiang Radiation Center, Hangzhou, China), with an LD_50_ of 426.7 Gy for Zhe 1613 and 318.3 Gy for Zhejing 100, applied at 60 Gy/h (^137^Cs source with 21,490 Ci and 80 cm distance). Control seeds were treated with 0 Gy.

### 4.2. BR Treatment and Seed Germination

The BR treatment consisted of two parts: pre-sowing soaking and post-sowing spraying. More specifically, three replicates of 200 randomly selected irradiated seeds were soaked in solutions with BR concentrations of 0.001, 0.01, 0.1, 1.0, and 5.0 μmol/L (designated as C1, C2, C3, C4, and C5, respectively) for 24 h in a room with a temperature of approximately 28 °C. Control seeds were soaked in distilled water (i.e., CK). The seed-to-solution ratio was 1:3 (*w*/*v*). The soaked seeds were placed in Petri dishes containing three layers of moistened filter paper and then incubated in darkness for 48 h under controlled conditions (28 ± 1 °C and 80% humidity) to promote germination. For each treatment, 192 incubated seeds were transferred to two 96-hole (8 lines and 12 holes per line) plastic hydroponic pots (length 12.6 cm × width 8.6 cm × height 11.4 cm; Nantong Experimental Equipment Co., Ltd., Nantong, China; Figure S4) filled with 0.9 L Yoshida nutrient solution (pH 5.8) (Yoshida et al., 1976 and Thakur et al., 2020) [38,39]. The nutrient solution was replaced every 5 days. At 7–14 days after the seeds germinated, the seedlings were sprayed twice with a 0.1 μmol/L BR solution (at 8:00 pm on each treatment day, with 2 mL per 96-hole pot each time). Control seedlings were sprayed with distilled water (same treatment time and volume). The complete treatments consisting of post-sowing spraying and a pre-sowing soaking in solutions with different BR concentrations were designated as C1pt, C2pt, C3pt, C4pt, and C5pt. The seedlings in plastic hydroponic pots were incubated in KBW400 growth chambers (Binder GmbH, Tuttlingen, Germany) set at 28 ± 1 °C, with a 12 h light (photosynthetic photon flux density of 360 μmol m^−2^ s^−1^)/12 h dark cycle and 75% ± 5% relative humidity. After 21 days, 50 living seedlings were collected for the growth parameter and physiological indices assay. The remaining seedlings were used to calculate the survival rate.

### 4.3. Seedling Emergence and Survival Rate Test

Seeds were considered to have germinated if the embryo broke through the seed coat and then extended by at least 2 mm (Lu et al., 2024) [14]. The seed germination rate (%) was determined at 7 days after sowing by counting the number of seedlings in the hydroponic pots. Seedlings with normal roots (i.e., white) and shoots were considered to be healthy. The seedling survival rate was calculated on the basis of the number of healthy seedlings at 3, 7, 14, and 28 days after sowing. Moldy, non-germinated seeds and seedlings were removed daily.

### 4.4. Seedling Growth Parameter Test

Seedling shoot and root fresh weight and dry weight were measured using an electronic balance (Sartorius, Shanghai, China), with 40 plants as one group from each replicate. The average weights were recorded.

### 4.5. Determination of Chlorophyll and Carotenoid Contents

To determine the chlorophyll and carotenoid contents, 0.1 g of frozen shoot samples were homogenized with 10 mL of acetone (80% *v*/*v*) using a pre-cooled pestle and mortar. Then, the homogenate was centrifuged at 5000× *g* for 10 min. The absorbance was measured with a UV–visible spectrophotometer (Beckman Coulter, Brea, CA, USA) at 640 nm and 663 nm for the estimation of total chlorophyll and 470 nm for total carotenoid content. The chlorophyll contents were calculated using the equations proposed by Lalarukh et al. (2022) [40]. The contents were expressed as mg g^−1^ fresh weight.

### 4.6. Determination of O_2_^•−^, H_2_O_2_, and MDA Contents

The O_2_^•−^ content was measured as described by Achary et al. (2012) [41] and Li et al. (2018) [42]. Fresh shoots (0.1 g) were immersed in a solution containing 50 mM Tris-HCl buffer (pH 6.4), 0.2 mM nitroblue tetrazolium, 0.2 mM reduced nicotinamide adenine dinucleotide, and 250 mM sucrose prior to the 10–15 min vacuum infiltration. The shoots were kept in the solution and irradiated (200 mol m^−2^ s^−1^) for 24 h. The absorbance at 530 nm was recorded.

The H_2_O_2_ levels were determined as described by Lin et al. (1988) [43]. A fresh shoot sample (0.1 g) was homogenized in 3 mL 50 mM phosphate buffer (pH 6.5). The homogenate was centrifuged at 6000× *g* for 25 min. A 3 mL aliquot of the extracted solution was mixed with 1 mL 0.1% titanium sulfate in 20% (*v*/*v*) H_2_SO_4_. The mixture was centrifuged at 6000× *g* for 15 min. The intensity of the yellow coloration of the supernatant was measured at 410 nm. Absorbance values were calibrated on the basis of a standard curve generated using known H_2_O_2_ concentrations. The H_2_O_2_ contents were expressed in terms of μmol g^−1^ fresh weight.

The MDA level was measured using thiobarbituric acid as described by Wu et al. (2003) [44]. A fresh shoot sample (0.1g) was homogenized in 4 mL 0.1% (*w*/*v*) trichloroacetic acid in an ice bath. The homogenate was centrifuged at 12,000× *g* for 20 min, and the supernatant was retained for the lipid peroxidation analysis. A 1 mL aliquot of the supernatant was mixed with 4 mL of 0.5% thiobarbituric acid in 20% trichloroacetic acid. The mixture was incubated in boiling water for 30 min. The MDA content was then determined according to the spectrophotometric absorbance at 532 nm and corrected for nonspecific turbidity at 600 nm.

### 4.7. Determination of Proline, AsA, GSH, and GSSG Contents

The proline content was determined as described by Shin et al. (2021) [45]. Approximately 0.1 g fresh shoot sample was treated with 2-hydroxy-5-sulfobenzoic acid, and then the homogenate was centrifuged at 5000× *g* for 10 min. Acid ninhydrin and glacial acetic acid were added to 1 mL supernatant. The resulting mixture was heated at 95 °C for 1 h. The reaction was stopped using an ice bath, and then toluene was added to the solution. The absorbance at 520 nm was recorded.

Fresh shoot samples (0.1 g) were ground in 5 mL 10% (*w*/*v*) trichloroacetic acid at 2 °C and centrifuged at 15,000× *g* for 10 min. The supernatant was used to determine the GSSG, total glutathione, and GSH contents. The AsA content was measured as described by Hodges et al. (1996) [46]. The GSSG and total glutathione contents were measured as described by Griffith (1980) [47]. The GSSG content was determined after GSH was removed via a 2-vinylpyridine derivatization. The GSH content was then estimated on the basis of the difference between the total glutathione and GSSG contents. A standard curve prepared using GSH and GSSG was used to calculate the total glutathione, GSH, and GSSG contents.

### 4.8. Determination of Antioxidant Enzyme Activities

The total SOD activity was determined as described by Prochazkova et al. (2001) [48] and Lu et al. (2024) [14]. Shoot samples (0.5 g fresh weight) were ground in 8 mL extraction buffer using a pestle and an ice-cold mortar, as described by Lu et al. (2024) [14]. The 50 mM phosphate buffer (pH 7.8) used to extract SOD contained 0.1 mM ethylenediaminetetraacetic acid (EDTA), 0.5% (*m*/*v*) polyvinylpyrrolidone, and 0.1% Triton X-100. The homogenates were filtered through four layers of gauze and then centrifuged at 12,000× *g* for 20 min at 4 °C. The supernatants were collected for the analysis of antioxidant enzyme activities. The 50 mM phosphate buffer (pH 7.8) used to extract CAT contained 1% (*m*/*v*) polyvinylpyrrolidone, 0.1% Triton X-100, and 0.1 mM EDTA. Peroxidase (POD) activity was assayed as described by Putter (1974) [49], with some modifications. The reaction mixture consisted of 100 μL enzyme extract, 100 μL guaiacol (1.5%, *v*/*v*), 100 μL 300 mM H_2_O_2_, and 2.7 mL 25 mM potassium phosphate buffer supplemented with 2 mM EDTA (pH 7.0). The APX activity was determined by monitoring the decrease in the absorbance at 290 nm, as described by Nakano and Asada (1981) [50]. The assay mixture comprised 100 μL enzyme extract, 100 μL 7.5 mM AsA, 100 μL 300 mM H_2_O_2_, and 2.7 mL 25 mM phosphate buffer (pH 7.0).

### 4.9. Statistical Analysis

The experiment was laid out in factorial randomized block design (FRBD) with three replications. A total of 192 incubated seeds were planted in two 96-hole plastic hydroponic pots and were considered one replication. Critical difference (CD) at 5% level of significance was used to compare the differences between the treatments. Analysis was performed through the IBM SPSS Statistics 21.0 (SPSS Inc., Chicago, IL, USA) application, and Duncan’s multiple range test was used to compare treatment mean.

## Figures and Tables

**Table 1:** Effects of soaking rice seeds in solutions with different BR concentrations on the germination rate.

Treatments	CK	C1	C2	C3	C4	C5
Zhe 1613	94.7 ± 1.2 a	95.7 ± 1.5 a	95.3 ± 3.5 a	95.7 ± 1.5 a	94.0 ± 1.0 a	87.7 ± 2.5 b
Zhejing 100	85.0 ± 3.0 a	84.3 ± 3.1 a	88.3 ± 3.1 a	87.0 ± 3.6 a	86.0 ± 4.4 a	87.7 ± 4.9 b

Note: The seed germination rate was determined at 7 days after sowing. Data are presented as the mean ± standard error (*n* = 3, three replicates and 192 seeds for each replicate). Different lowercase letters in each column indicate a significant difference at the 0.05 level (Duncan’s multiple range test).

**Table 2:** Effects of different BR treatment combinations on the survival rate of irradiated rice seedlings.

Treatments	CK	C1pt	C2pt	C3pt	C4pt	C5pt
Zhe 1613	49.7 ± 3.2 c	56.0 ± 2.6 b	63.3 ± 3.5 a	61.7 ± 2.9 a	55.7 ± 2.5 b	47.7 ± 3.5 c
Zhejing 100	46.7 ± 2.5 c	52.7 ± 1.5 b	59.3 ± 4.2 a	58.0 ± 2.6 a	53.0 ± 1.7 b	43.7 ± 3.2 c

Note: The seedling survival rate was determined at 28 days after sowing. Data are presented as the mean ± standard error (*n* = 3). Different lowercase letters in each column indicate a significant difference at the 0.05 level (Duncan’s multiple range test).

## Data Availability

The raw data supporting the conclusions of this article will be made available by the authors without undue reservation.

## Supplementary Materials

The supporting information can be downloaded at: https://www.mdpi.com/article/10.3390/ijms252111523/s1.
