# Supplementary material for: Exogenous Brassinolide Ameliorates the Adverse Effects of Gamma Radiation Stress and Increases the Survival Rate of Rice Seedlings by Modulating Antioxidant Metabolism
Source: Int J Mol Sci. 2024 Oct 26;25(21):11523. doi: 10.3390/ijms252111523 (PMC11546918; doi:10.3390/ijms252111523)
Supplement: Supplementary file 1 [file ijms-25-11523-s001.zip › ijms-3219735-supplementary.pdf]

Supplementary materials:

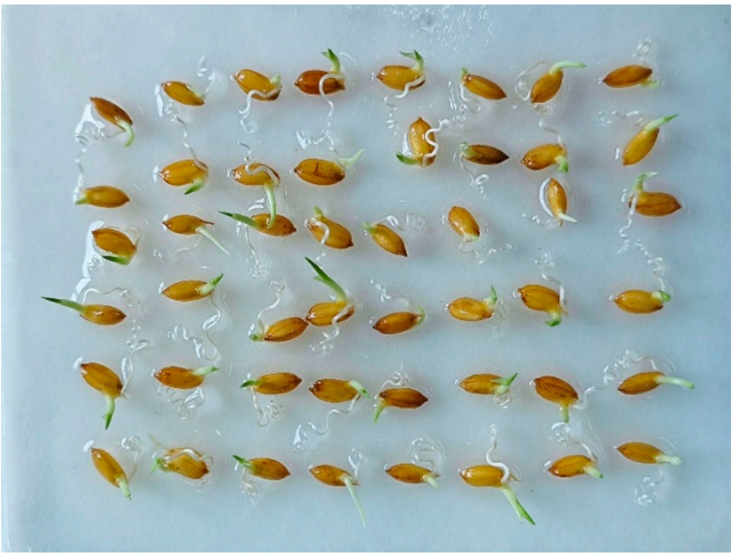

Figure S1. The effect of high concentration BR solution soaking on γ ray irradiated rice seed germination, seedling emergence.

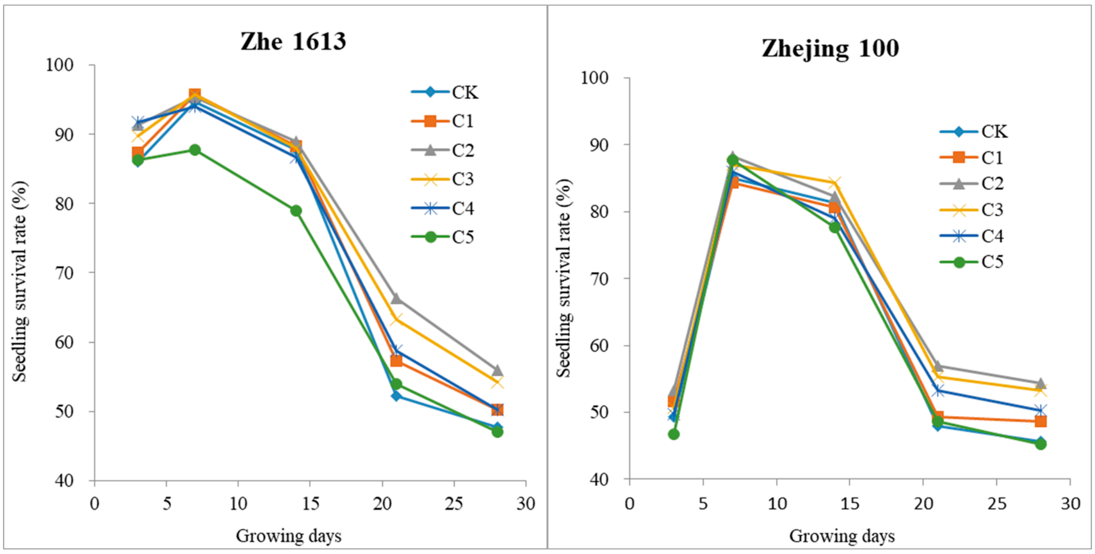

Figure S2. The effect of different concentration BR solution soaking on γ ray irradiated rice seedling survival rate.

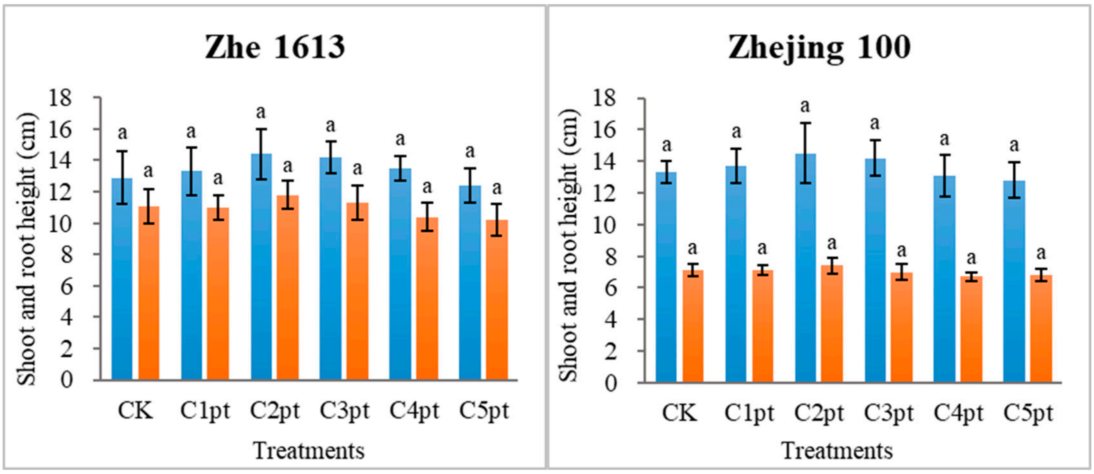

Figure S3. The effect of different concentration BR solution soaking on γ ray irradiated rice seedling shoot and root height.

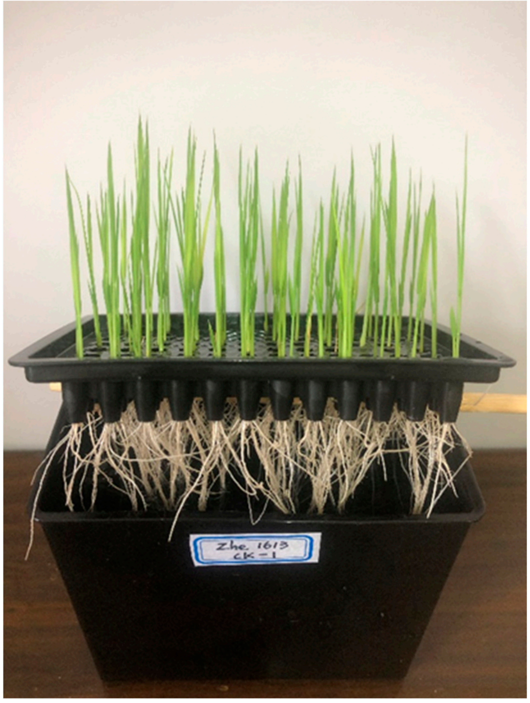

Figure S4. The plastic hydroponics pot for rice seedling culture.

Table S1. Effects of different BR concentration solution soaking on the survival rate of irradiated rice seedlings

Treatments	CK	C1	C2	C3	C4	C5
Zhe 1613	47.7±3.5 c	50.3±2.3 bc	56.0±2.6 a	54.3±1.5 ab	50.3±2.1 bc	47.0±3.0 c
Zhejing 100	45.7±2.5 c	48.7±2.1 bc	54.3±2.5 a	53.3±2.1 a	50.3±3.1 ab	45.3±2.1 c

Note: The seed germination rate was determined at 7days after planting. Data are means±standard error (*n*=3). The lowercase letters in each column represent significance at the 0.05 levels (Duncan's multiple range test).
